# Systemic polidocanol from intravenous or pressurized intrauterine administration produces reversible cardiovascular toxicity

**DOI:** 10.1016/j.jvssci.2022.08.002

**Published:** 2022-10-20

**Authors:** Jeffrey T. Jensen, Philberta Leung, Mackenzie Roberts, Jian Guo, Shan Yao, Emily Mishler, Tanner Grenz, James Hodovan, Ov D. Slayden, Jonathan R. Lindner

**Affiliations:** a Department of Obstetrics & Gynecology, Oregon Health & Science University (OHSU), Portland, OR; b Division of Reproductive and Developmental Sciences, Oregon National Primate Research Center, Beaverton, OR; c Center for Regenerative Medicine, Oregon Health & Science University (OHSU), Portland, OR; d Knight Cardiovascular Institute, Oregon Health & Science University (OHSU), Portland, OR; e Division of Cardiometabolic Health, Oregon National Primate Research Center, Beaverton, OR

**Keywords:** Sclerosis, Polidocanol, Ventricular function, Primates, Endometrium

## Abstract

**Objective:**

Fatal allergic responses and cardiac arrhythmias have been reported with the intravenous (IV) administration of polidocanol. We sought to identify the physiologic mechanism of systemic cardiovascular response after transcervical (TC) and IV administration of polidocanol.

**Methods:**

We continuously monitored blood pressure (BP) and heart rate using an arterial line during IV and intraperitoneal (IP) administration of polidocanol solution (PS) and polidocanol doxycycline solution in female rats and TC and IP administration of polidocanol foam (PF) and PDF (TC only) in female baboons. We performed TC procedures using a catheter with (pressurized) and without (nonpressurized) balloon inflation. Baboons also underwent monitoring during IV PS administration with and without pretreatment with antihistamines. We performed cardiac echo and electrocardiograms during selected experiments. We defined a refractory hypotension as a sustained decrease of more than 30% from baseline that prevented delivery of the target dose.

**Results:**

We found a dose-related increase in the proportion of baboons that developed refractory hypotension during TC administration of 5% PDF and PF, an effect confined to pressurized administration. The infusion of 0.5% PS in rats induced a rapid and dramatic refractory hypotension. The inclusion of doxycycline did not improve or deteriorate these outcomes, and doxycycline solution or saline (control) alone did not affect BP. All five female baboons that received up to 20 mL of 1% PS (200 mg) developed refractory hypotension. Pretreatment with diphenhydramine, ranitidine, or both did not block the refractory hypotension induced by IV administration of 1% PS (100 mg). In contrast, only one of the six female baboons treated with IP PF 400 mg developed a decrease of more than 30% in BP, and this response was not sustained. Cardiac echocardiography done in four baboons during TC treatment demonstrated a decrease in cardiac output as the physiologic mechanism of hypotension. We did not observe important changes on the electrocardiograms.

**Conclusions:**

Adverse cardiovascular effects of polidocanol treatment occur owing to a direct myocardial effect of polidocanol and not as a result of a hypersensitivity reaction. Pressurized TC administration of PF results in refractory hypotension owing to endometrial vascular uptake of polidocanol and not as a result of uptake from peritoneal surfaces.

## Clinical Relevance

Our experiments in rodent and nonhuman primates demonstrate that hypotension during polidocanol treatment occurs as a consequence of low cardiac output secondary to impaired biventricular function. The onset is acute, dose dependent, and precipitous, and resolves slowly upon discontinuation of the polidocanol infusion with appropriate supportive care. The physiologic features of this response are not explained by classical allergy, or by a complement-mediated pseudoallergic response. We propose that many of the adverse events reported after the therapeutic use of PS and PF occur owing to this direct effect on both the pulmonary vasculature and the myocardium and not as an allergic mechanism. Further investigations of this response mechanism may provide insight into potential therapeutic interventions to improve safety of polidocanol treatment.

**Article Highlights**

• **Type of Research:** Rodent and nonhuman primate models
• **Key Findings:** Direct intravenous and pressurized intrauterine administration of polidocanol causes adverse cardiovascular effects through a direct effect on the myometrium that results in a decrease in cardiac output. The effects are dose dependent.
• **Take Home Message:** Adverse cardiovascular events that occur after the clinical use of polidocanol likely result from a direct cardiac, and not allergic, mechanism.

The intravenous (IV) administration of a sclerosing agent provides a nonsurgical approach to the management of a variety of chronic vascular diseases including telangiectasias, reticular veins, and varicose veins.^1^ Detergent surfactants like polidocanol have regulatory approval in many countries as injectable solutions and foams for venous sclerosis.^1^ Sclerosing agents are also used off-label for other indications, including sclerosis of bone,^2^ renal,^3^ hepatic,^4^ and lymphatic cysts.^5^ Sclerosing agents in clinical use differ with respect to mechanism of action and toxicity profiles.^6^ They act by damaging the endothelium or epithelium; this damage results in collapse of the vessel or cyst followed by inflammation and collagen occlusion during repair.

Local tissue reactions at the injection site are the most common adverse events observed with injectable sclerosing agents.^6^ Although serious adverse effects are uncommon, a recent analysis of data from the World Health Organization pharmacovigilance database evaluated reports of six adverse event syndromes of interest: hypersensitivity reactions, arterial thromboembolic disorders, venous thromboembolic disorders, cardiac arrhythmias, visual/neurological disturbances, and skin ulcerations.^7^ Fatal allergic responses and cardiac arrhythmias have been reported for all sclerosing agents, including polidocanol.^7^, ^8^, ^9^

Type I hypersensitivity responses develop when specific IgE antibodies, induced by a prior exposure to an antigen, cross-link and trigger the release of histamine and other inflammatory mediators upon re-exposure to the antigen.^10^ Type II hypersensitivity reactions develop when IgG or IgM antibodies recognize cell surface or extracellular matrix antigens and bind the Fc receptor of macrophages or activate the complement pathway directly.^11^ Szebeni^12^ proposed the term complement activation-related pseudoallergy (CARPA, or type III hypersensitivity) to differentiate hypersensitivity responses that activate complement independent of antibodies and mimic true allergic anaphylaxis pathways associated with first-time exposures to certain drugs and contrast agents.^12^ Because both true allergic responses and CARPA require the activation of a histamine receptor, strategies to prevent CARPA include the prophylactic administration of histamine receptor antagonists.^12^

We have conducted early investigations in nonhuman primates evaluating the safety and efficacy of polidocanol (PF) and polidocanol doxycycline (PDF) foam sclerosis of the fallopian tube for the indication of nonsurgical permanent contraception.^13^, ^14^, ^15^, ^16^ Doxycycline has independent activity as a sclerosing agent,^17^ so we tested PDF to determine if the combination could improve the rate of tubal occlusion observed with PF alone. We administer PF or PDF transcervically (TC) into the uterine cavity using a balloon catheter to occlude the cervix. Foam must fill the cavity before spilling out the fallopian tubes. In the baboon model, the small diameter fallopian tubes (approximately 100 microns) generate considerable resistance to flow that results in the pressurization of foam within the endometrial cavity. We discovered that some animals developed refractory hypotension, suggestive of severe anaphylaxis, during TC foam treatment, with findings similar to those previously reported during vascular sclerosis procedures with polidocanol.^18^ This finding led us to conduct a series of experiments to mechanistically explain the physiologic basis for the adverse cardiovascular response to polidocanol treatment.

## Methods

### Animal care

The animals used in these experiments include not only nonhuman primates, but also rats, which makes it possible to increase the size of animal numbers for different exploratory approaches because of the greater availability of small animals for in vivo study.

The Institutional Animal Care and Use Committees (IACUC) at the Oregon National Primate Research Center (ONPRC) and Kenya Institute of Primate Research approved all of the baboon procedures at their respective institutions. Animal husbandry provided by both institutions is in accord with the National Institutes of Health Guidelines for the Care and Use of Laboratory Animals,^19^ with all animals maintained in social housing groups. We conducted all of the surgical procedures under general anesthesia using IACUC-approved protocols. Adult female baboons (*Papio anubis*, *Papio hamadryus*) aged 12 ± 7 years and weighing 17 ± 8 kg used in the study underwent sedation with ketamine (10 mg/kg IM; Putney, Dublin, OH) before endotracheal intubation and induction and maintenance of general anesthesia with isoflurane (WI, Boise, ID) inhalation (see the Supplementary materials for additional details).

Rodent studies received IACUC approval at ONPRC. We obtained healthy adult (250-360 g) female Sprague-Dawley rats from a vendor (Charles River Laboratories, Wilmington, MA). Trained veterinary staff performed all of the procedures under mask general anesthesia with isoflurane.

### Preparation of polidocanol solution and foam

We obtained bulk polidocanol from two different commercial sources, Sigma (St Louis, MO) (research grade) and LGM Pharma (Boca Raton, FL) (pharmaceutical grade). Detailed analyses comparing the two sources showed no important chemical differences (data not shown). We prepared a polidocanol solution (PS) (weight/volume) by warming bulk polidocanol to 37°C for 10 minutes before dilution with Dulbecco’s phosphate-buffered saline (DPBS) to the required volume. A 5% PS contains 50 mg of polidocanol in 1 mL of DPBS: a 1% solution contained 10 mg/mL, and a 0.5% solution contains 5 mg/mL. To prepare polidocanol foam (PF), we draw 1 mL of the PS (1 mL) into a 5-mL syringe, and fill a second 5-mL syringe with 4 mL of air. We then used a modified Tessari technique^20^ to generate foam by passing the air and solution mixture back and forth 20 times through a 5-μm syringe filter connected between the two syringes with Luer lock fittings. On the final pass, this process results in 5 mL of foam collected in one syringe for injection. The process is repeated for subsequent 5-mL aliquots. Some animals received a combined polidocanol/doxycycline solution or foam (PDF). To prepare this, we added 100 mg of doxycycline powder (Novaplus, Fresenius Kabi, Lake Zurich, IL) to 4 mL of PS, agitating the solution until dissolved before generating foam.

### IV and intraperitoneal administration of PS in rats

We used rats to compare the (1) cardiovascular effects of different PS (eg, research grade or pharmaceutical grade; doxycycline or no doxycycline) when administered IV and (2) cardiovascular effects of the same dose of PS administered IV and intraperitoneally (IP). We used solutions rather than foams in these experiments to eliminate the potential confounding factor of air embolism. We evaluated doxycycline, because we sought to determine if this agent changed the cardiovascular effects of polidocanol. To minimize the number of animals used, data from some rats were used in multiple comparisons.

Anesthetized females underwent dissection of the femoral triangle to provide exposure for placement of a 2F polyurethane medical tubing into the femoral vein for IV access, and another catheter into the femoral artery for continuous monitoring of blood pressure (BP). For IP infusions, we placed an 18G angiocath directly through the abdominal wall into the peritoneal cavity. We used a syringe pump to infuse all test solutions IV at a rate of 0.1 mL/min through the femoral vein. All of the experiments used the same sequence: (1) baseline (saline for 3 minutes [0.3 mL]); challenge 1 (test agent—polidocanol, doxycycline, or combination for 5 minutes [0.5 mL]); recovery 1 (no infusion for up to 7 minutes [minimum of 3 minutes if no change from baseline during challenge 1); Challenge 2 (same test agent as Challenge 1 for 2 minutes [0.2 mL]); and recovery 2 (5-20 minutes).

Rats (n = 6 per group) received one of the following IV test agents: (1) PS 0.5% (research grade source); (2) PS 0.5% (pharmaceutical grade source); (3) Asclara (US Food and Drug Administration-approved 0.5% PS product); (4) PS 0.5% with a doxycycline 25 mg/mL solution (polidocanol doxycycline) (research grade source); (5) polidocanol doxycycline (pharmaceutical grade source); (6) doxycycline solution (25 mg/mL saline); or (7) DPBS (control). Active treatments provided 3.5 mg polidocanol and/or 17.5 mg doxycycline. Rats undergoing IP administration received only PS 0.5% (pharmaceutical grade source).

### TC administration of PF in baboons

We performed cervical dilation, placed a 7F silicone hysterosalpingogram (HSG) balloon catheter (J-CHSG-703000; Cook, Bloomington, IN) into the uterine cavity of female baboons under ultrasound guidance, evaluated tubal patency, and infused PF through the catheter into the uterine cavity as previously described.^16^ Inflation of the balloon of the HSG catheter in the lower uterine segment seals the cavity and prevents leakage of the administered agent through the cervix as uterine pressure increases to a level sufficient to overcome the resistance of flow for movement of the solution or foam into the fallopian tubes. TC administration of the solution or foam without balloon inflation allows leakage through the cervix and prevents uterine cavity pressurization and tubal delivery.

To evaluate the effect of polidocanol dose, we performed TC administration of PDF with the HCG balloon inflated (pressurized) in one baboon group (n = 9). Among these animals, five received a target dose of 20 mL (200 mg polidocanol) and four a target dose of 40 mL (400 mg). We discontinued foam administration upon reaching a prespecified stopping point of a 30% decrease from baseline in mean arterial pressure (MAP). Animals underwent electrocardiograms (ECG) during these procedures.

We then performed a controlled experiment in baboons to directly compare the effects of pressurized and nonpressurized foam delivery. Females (n = 5) received 5% PF using two different routes of administration in the following sequence: (1) TC nonpressurized (eg, Cook HSG catheter placed without inflation of balloon) and (2) TC pressurized (eg, Cook HSG catheter placed with inflation of balloon). We conducted the two treatments approximately 1 month apart at the same menstrual cycle phase (peak tumescence) to control for hormonal effects.^16^^,^^21^ The females received up to 40 mL of 5% PF (400 mg polidocanol) during each procedure. We discontinued the foam administration upon reaching a prespecified stopping point of a 30% decrease from baseline in MAP.

The same investigator performed all of the PDF and PF infusions, delivering foam by hand at an approximate rate of 2 mL/minute.

### IP administration of PF

In addition to uptake from endometrial blood vessels, polidocanol could enter the vascular space by direct absorption from peritoneal surfaces after spillage from the fallopian tubes during TC procedures. To determine whether clinically important cardiovascular effects result from peritoneal uptake, we performed direct IP injection of polidocanol. We used two approaches for IP delivery, namely, laparoscopic and direct injection. For laparoscopic delivery, anesthetized female baboons prepared for cardiovascular monitoring underwent insertion of a Cook HSG catheter through an accessory port with placement of the tip into the pouch of Douglas under direct visualization. After allowing the CO_2_ pneumoperitoneum to evacuate spontaneously, the investigator administered up to 40 mL (400 mg) of 5% PF through the catheter in 5-mL aliquots (approximately 2 mL/min). The catheter was then removed, and incisions closed. Other anesthetized females underwent blind placement of an 18G angiocath through the anterior abdominal wall, with direct delivery of the same dose.

### IV administration of PS and antihistamines in baboons

Because TC administration of PF results in unpredictable vascular uptake of polidocanol and introduces air bubbles as an additional variable, we delivered polidocanol as an IV infusion to study the effects of direct vascular administration with a standardized polidocanol exposure. For these experiments conducted at ONPRC, female baboons (n = 6, 14.9-16.1 kg) underwent placement of IV (peripheral vein of the arm) and arterial (posterior tibial) catheters under general anesthesia. After establishing baseline assessments, we used a syringe pump to administer 1% PS IV (pharmaceutical grade) at a rate of 1mL/min (10 mg/min) up to 200 mg (20 mL), with continuous measurement of BP and heart rate (HR) through an arterial line, and repeated record of end tidal CO_2_, respiratory rate, and oxygen saturation (O_2_ sat) every 1 minute during treatment, and then every 5 minutes during recovery (minimum 30 minutes). A 12-lead ECG was completed every 1 to 2 minutes.

We then used the same approach in baboons (n = 12) at Kenya IPR to evaluate whether histamine receptor blockade could prevent cardiovascular effects of polidocanol. After induction of general anesthesia, females received IV diphenhydramine (Mylan Pharmaceuticals, Canonsburg, PA), 25 mg; an H1 blocker (H1B), ranitidine (Zydus Pharmaceuticals, Pennington, NJ) 25 mg; an H2 blocker (H2B); both diphenhydramine and ranitidine (H1B and H2B); or no additional pretreatment (controls). After stabilization of the BP, we used a syringe pump to administer 1% PS IV at a rate of 1 mL/min (10 mg/min) and a total dose of up to 200 mg (20 mL) recording data as described elsewhere in this article.

### Cardiovascular and uterine pressure monitoring

During all of the experiments, we established baseline data for at least 10 minutes and then monitored animals continuously during administration of foam or solution and for at least 30 minutes after the infusion.

#### Invasive BP monitoring

We used a multichannel pressure monitoring system (ADInstruments, Colorado Springs, CO) to continuously record data during all of the procedures. The system provides continuous recording of HR, systolic BP (SBP), diastolic BP (DBP), and MAP through a single channel. For baboons, we placed a 25G catheter in the perineal artery, and connected this to the pressure monitoring system using an in-line pressure transducer (Deltran, Utah Medical, Midvale, UT). For rats, we used a 2F polyurethane medical tubing to cannulate the femoral artery and connected this to the Deltran transducer using a Luer adapter. During baboon experiments, we used the additional channels and Deltran transducers to simultaneously record intrauterine pressure (using a 2F polyurethane catheter placed within the uterine cavity) and pressure within the delivery catheter.

#### Noninvasive cardiovascular monitoring

During baboon procedures performed in the surgical suites at ONPRC, we recorded a number of parameters using the available Anesthesia monitoring equipment (Digicare Biomedical Technology, Inc., Boynton Beach, FL). These included end-tidal CO_2_ (EtCO_2_), respiratory rate, and O_2_ sat. We also recorded noninvasive measurements of SBP and DBP using a pediatric cuff. During the baboon PDF TC infusions only, we collected 12-lead ECGs (Cardio7.2, Bionet Co, Tustin, CA) at baseline and every 5 minutes throughout treatment and during recovery. For experiments conducted at Kenya IPR and for some baboon and all rat procedures at ONPRC, we used portable monitors for O2 sat (Nonin PalmSAT 2500 Digital Hand-Held Pulse Oximeter) and EtCO_2_ (Masimo EMMA Mainstream Capnograph Kit). We did not collect data on EtCO_2_ in rats.

#### Echocardiograms

An imaging team blinded to treatment-related variables performed transthoracic echocardiography (Vivid E95, GE Health Care, Chicago, IL) during selected TC baboon procedures at ONPRC at the following stages: stage 1, baseline; stage 2, upon completion of first 100-mg polidocanol administration; stage 3, upon completion of the peak dose of polidocanol; stage 4, 15 minutes after completion of administration; and stage 5, 25 after completion of administration. All measurements followed guidelines for adult echocardiographic measurement from the American Society of Echocardiography.^22^ Systemic vascular resistance (SVR) was calculated as the difference between the MAP from BP measurement and the right atrial pressure from inferior vena cava diameter and respirophasic variation, divided by the cardiac output derived from the product of HR and stroke volume by left ventricular (LV) outflow tract diameter-time velocity integral product. An index of pulmonary vascular resistance (PVR) was calculated without estimation of left-sided filling pressures by dividing the pulmonary artery peak systolic pressure (derived from tricuspid regurgitation jet velocity and inferior vena cava measurements) by the cardiac output. Peak contrast enhancement within the ventricular cavities, when observed after injection of sclerosing agents, was quantified by imaging with low-mechanical index (0.14) power modulation imaging at a dynamic range of 50 dB. Video intensity was measured from regions of interest in the ventricular cavities. Because treatments varied between animals, all data represent animal-specific findings.

#### Intrauterine pressure monitoring

To measure pressure within the uterine cavity during foam administration, we passed a 2F polyurethane medical tubing through the delivery channel of the HSG catheter. The tip extended 1 cm beyond the end of the catheter. We connected this to the Deltran transducer and recoding system using a Luer adapter.

### Data analysis

For IV administration of PS in baboons and rats, we calculated group means and standard deviations, and compared treatment time points using analysis of variance. For the TC experiments in baboons, we present individual data only without summary statistics or statistical comparisons owing to the variation in treatment response, and inability to make comparison between dose and techniques groups.

## Results

### Severe adverse effects of TC administration of PF in baboons are dose dependent and occur during administration with uterine cavity pressurization

**Fig 1:**
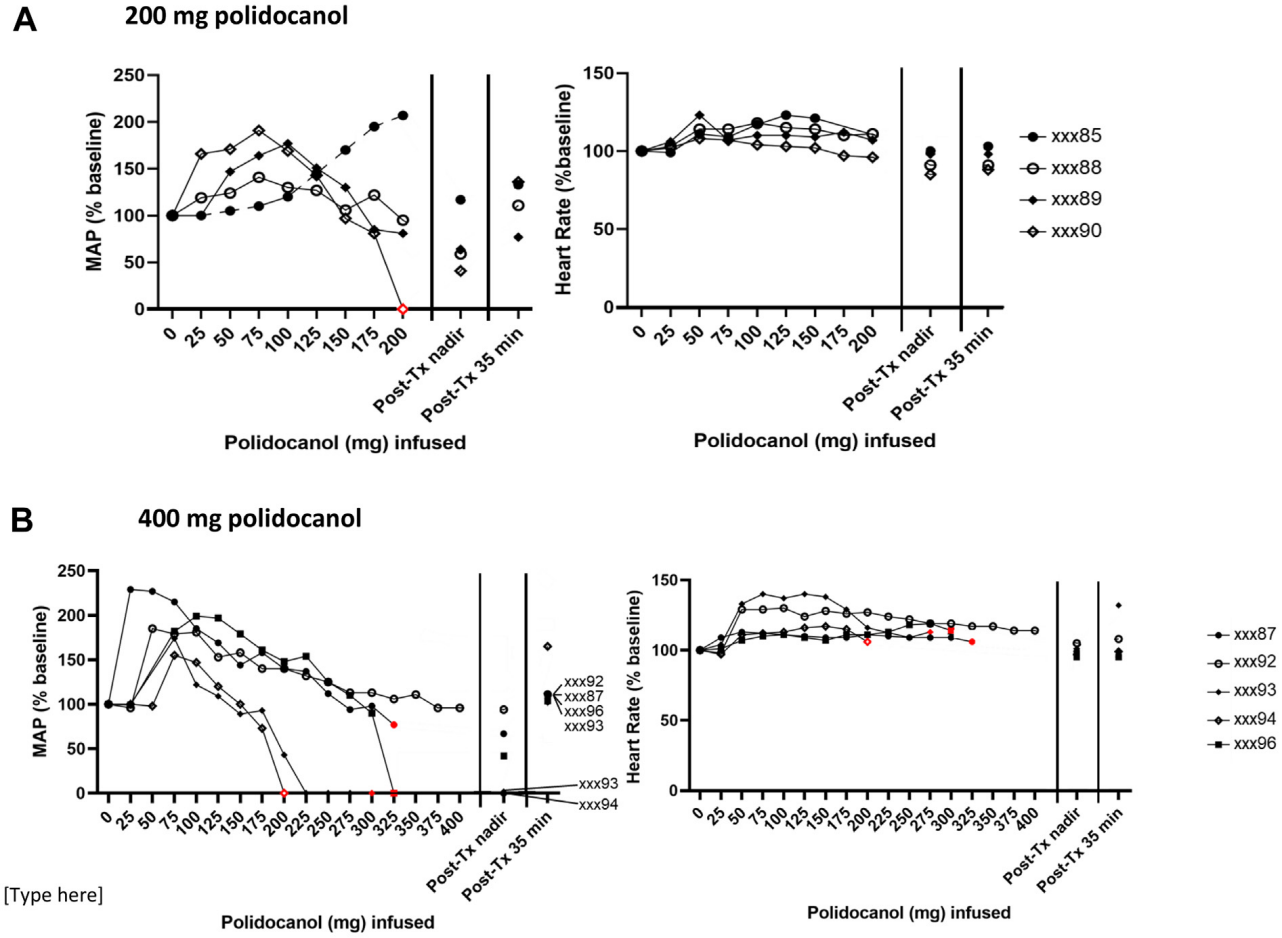
Mean arterial pressure (MAP) and heart rate (HR) in baboons in response to transcervical (TC) pressurized administration of polidocanol/doxycycline foam (PDF): effect of dose. MAP increased during administration of PDF, and then decreased below baseline for most animals, with cardiovascular collapse developing in some animals in the higher dose group. In contrast, HR remained stable or increased during PDF infusion, even with cardiovascular collapse developing in some animals in the higher dose group. **(A)** In the 200-mg group, all four animals tolerated the full target dose. Some data points extrapolated for xxx85 owing to infusion pump error (dotted line). Although we could not record a BP at the end of the polidocanol foam (PF) infusion in one female (xxx90), this resolved rapidly during recovery. **(B)** In the 400-mg group, only one of five tolerated the full target and two received epinephrine (xxx93, xxx94) for poor perfusion with several minutes of no data recorded. Data presented as percent of baseline. Blood pressure (BP) was presumed “0” if it was nonrecordable (red symbol in **A** and **B**). In **(B)**, red symbols also indicate when PDF delivery was prematurely terminated before reaching the target dose.

**Fig 2:**
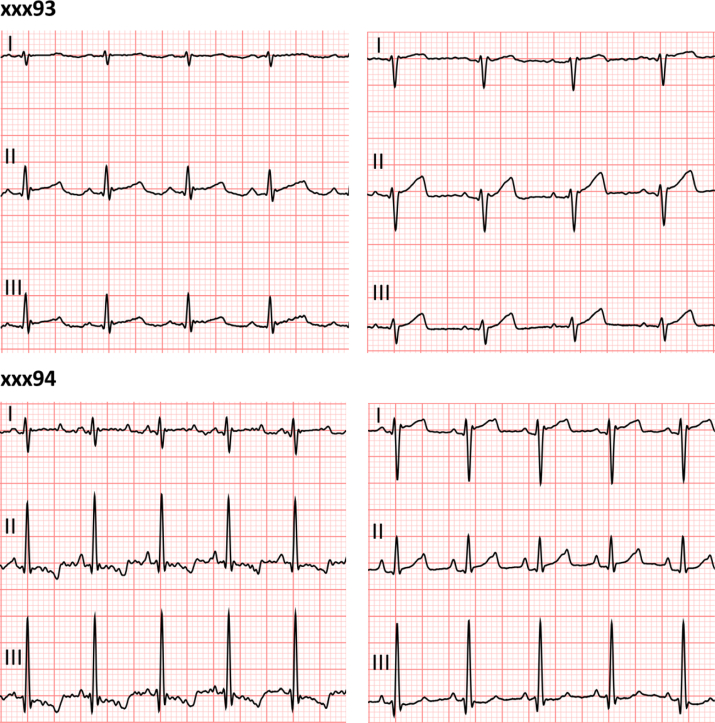
Representative electrocardiogram (ECG) changes during transcervical (TC) delivery of polidocanol foam (PF). Although baboons xxx93 (top) and xxx94 (bottom) exhibited refractory hypotension with no recordable blood pressure (BP) or pulse in response to PF infusion, we observed continued electrical activity on ECG throughout the procedure. (Left) Baseline ECG in leads I, II, and III before the start of the infusion. (Right) ECG in leads I, II, and III during episodes when no pulse or BP recorded with arterial line (pulseless electrical activity).

In the dose experiment, all four females in the 20-mL (200 mg polidocanol) PDF group received the full target dose. The SBP, DBP, and mean BP increased with the initiation of the PDF infusion, and this was followed by a steep decrease, with three of the four reaching a MAP nadir of less than 70% of baseline by the end of the infusion or during recovery (Fig 1, *A*, Supplementary Table I). All of the animals recovered within 30 minutes without further intervention. In contrast, four of the five animals in the 40-mL (400 mg) dose group experienced a treatment-ending decrease in BP before received the full target dose (Fig 1, *B*, Supplementary Table I), after developing a clinically important decrease in BP during the procedure. Two of these females received IV epinephrine (total 0.2 mg) for circulatory support, and the other two recovered without additional intervention. We did not observe clinically important changes in HR or O_2_ sat in any of the animals (Fig 1, Supplementary Tables II and III). However, we failed to record data with the pulse oximeter at critical time points in some animals, likely as a result of poor perfusion, because this occurred during episodes when we could also not record BP. However, the ECG showed pulseless electrical activity during these episodes of poor perfusion, ruling out asystole as the physiologic mechanism for the bradycardia (Fig 2).

**Fig 3:**
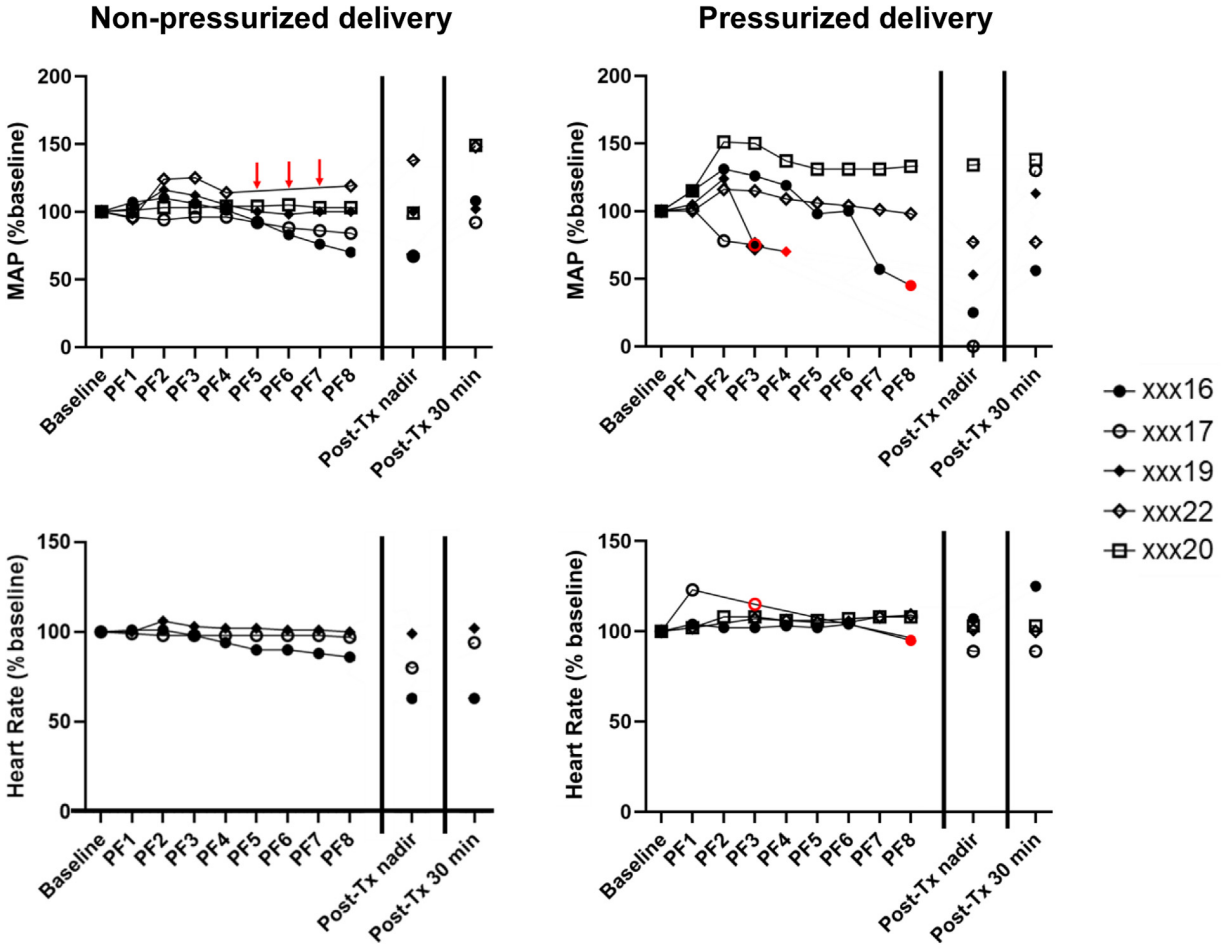
Effects of nonpressurized or pressurized transcervical (TC) delivery of polidocanol foam (PF) on cardiovascular effects. All five baboons tolerated the full target dose of 400 mg PF when administered without pressure (Cook HSG catheter inserted transcervically without balloon inflation). Although two baboons demonstrated a greater than 30% decrease in the mean arterial pressure (MAP) with nonpressurized delivery, this occurred near or after the completion of infusion and resolved rapidly without intervention. With pressurized PF delivery (Cook HSG catheter inserted transcervically with balloon inflation), three of five animals required premature termination of PF infusion and experienced a slow recovery from the induced hypotension. Heart rate (HR) and oxygen saturation (O_2_ sat) largely remained at baseline level throughout both PF infusion techniques (HR and O_2_ sat were not recorded for xxx20 and xxx22 during nonpressurized delivery or for xxx19 during pressurized delivery). Red arrows indicate missing data owing to a lack of signal from monitoring equipment. Red symbols indicate when PDF infusion was terminated before reaching the target dose. *Tx*, treatment.

**Fig 4:**
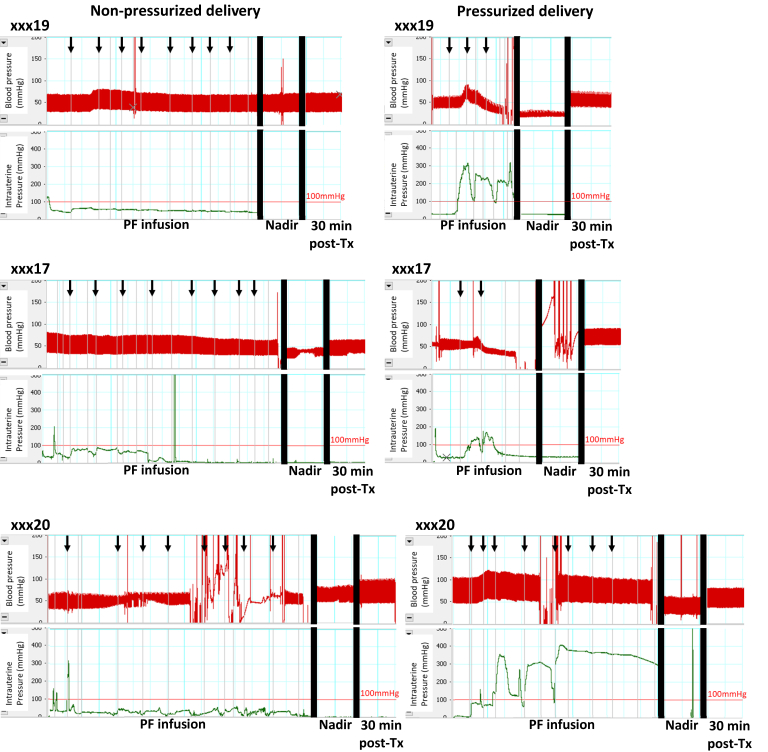
Representative continuous blood and intrauterine pressure records during nonpressurized and pressurized transcervical (TC) delivery of polidocanol foam (PF) in the same animals. Administration of PF without inflation of the HSG catheter balloon (left) does not result in uterine pressure above 100 mm Hg, a threshold exceeded during administration with balloon inflated (right). Animals tolerated the entire target dose of 400 mg of PF during nonpressurized delivery (left) without clinically important changes in blood pressure (BP) (eg, xxx19) or with mild and transient changes near the end of the infusion (eg, xxx17), but showed evidence of circulatory collapse during pressurized administration (eg, xxx19, xxx17) (right). Some animals were resistant to the effects of polidocanol; xxx20 developed only transient and rapidly reversible decrease in BP with pressurized foam delivery. All traces include the duration of PF administration, nadir of BP response during recovery, and 30 minutes after the end of treatment (breaks indicated by solid vertical black bars). Thin red line = reference line of 100 mm Hg for intrauterine pressure. Black arrows = start of infusion for each 5 mL PF (50 mg polidocanol each). *Tx*, treatment.

To compare the effect of uterine pressurization, five other female baboons underwent TC administration of up to 40 mL (400 mg) 5% PF through a Cook HSG catheter without and with inflation of the balloon. During the first procedure with nonpressurized foam, all five tolerated the full 400-mg dose of polidocanol. The mean uterine cavity pressure during foam infusion remained less than 100 mm Hg. Although two animals demonstrated a more than 30% decrease in MAP, this occurred only at or after completion of the foam infusion, and resolved rapidly without intervention. In contrast, the same five animals undergoing pressurized foam delivery (uterine pressure >150 mm Hg) experienced a more than 30% decrease in MAP during or after the infusion (Fig 3, Supplementary Table IV). The severity of this decrease required premature termination of the PF infusion before reaching the target dose in all four animals. Although all of the animals recovered without intervention, hypotension persisted for more than 30 minutes in most cases, with the magnitude of effect related to total dose received. The animal that tolerated the full 400-mg dose experienced only a transient decrease in MAP during recovery. Consistent with the dose experiment, we did not record important changes in HR or O_2_ sat in any of the animals with either treatment. Fig 4 provides actual details of the relationship between uterine pressure and BP during pressurized and nonpressurized PF delivery in representative animals. Treatment with pressurized PF resulted in a slight increase in BP in most animals, followed by a decrease in MAP associated with a narrowing of the pulse pressure. During recovery, the pulse pressure and MAP gradually increased. As expected, intrauterine pressure was greater during pressurized foam delivery, and this finding was associated with more significant changes in BP. We were unable to record intrauterine pressure during the nonpressurized treatment in baboon xxx16; the investigator subjectively reported high resistance during foam administration and did not note the expected spillage of foam through the cervix. This female experienced a more than 30% decrease at the end of treatment (Supplementary Table IV).

### IV administration of PS in rats results in reversible hypotension: Doxycycline has no effect

**Fig 5:**
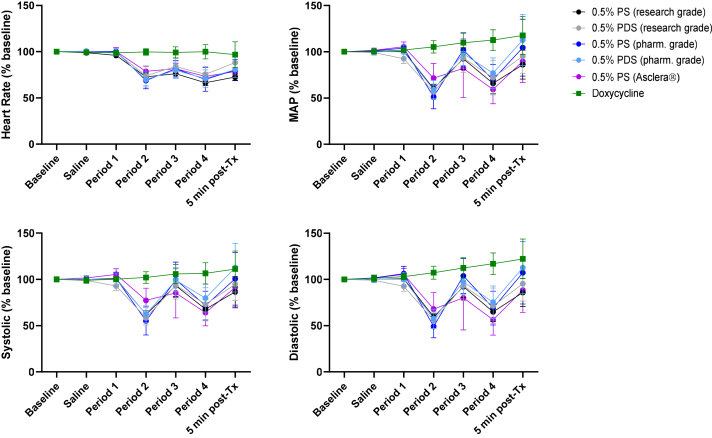
Change in blood pressure (BP) in rats after intravenous (IV) administration of PS, with or without doxycycline. After establishing a stable baseline, rats received the following sequence of experimental regimen: saline, saline for 3 minutes (0.3 mL); period 1, test agent for 5 minutes (0.5 mL); period 2, no infusion for 3-7 minutes; period 3, same test agent as in period 1 for 2 minutes (0.2 mL); and period 4, recovery for 5-20 minutes. Data in the figures represent the start of each period. Test agent solutions tested were polidocanol, doxycycline, or a combination of the two. Infusion of a 0.5% PS induced a rapid and drastic decrease in all monitored cardiovascular parameters. The inclusion of doxycycline did not improve or deteriorate the outcomes, and doxycycline solution alone did not affect BP. The changes were independent of polidocanol source. Rapid recovery was observed following each challenge. Data (normalized to baseline) presented as mean ± standard deviation (normalized to percent baseline). n = 4-6 per group. *MAP*, mean arterial pressure; *PDS*, polidocanol doxycycline solution; *PS*, polidocanol solution; *Tx*, treatment.

To compare the effects of PS with and without doxycycline directly, we performed experiments in rats using controlled IV administration to have sufficient animal numbers for statistical comparisons of the results and to eliminate potential confounding owing to air bubbles in foam. During the initial control infusion of saline, the HR and BP (SBP, DBP, MAP) did not change from baseline. In contrast, IV infusion of polidocanol, with or without doxycycline, resulted in a rapid decrease in both the BP and the HR, which recovered rapidly with no important differences between polidocanol sources (Fig 5). These effects of polidocanol were transient with recovery seen at 5 minutes. With most polidocanol treatments, BP changes reached or approached a 50% decrease from baseline and there was no worsening of the effect with repeat dosing. In contrast, the administration of doxycycline alone did not result in any effect on the BP or HR.

### Adverse cardiovascular effects occur with IV administration of PS in baboons, and antihistamine treatment does not mitigate or prevent these findings

**Fig 6:**
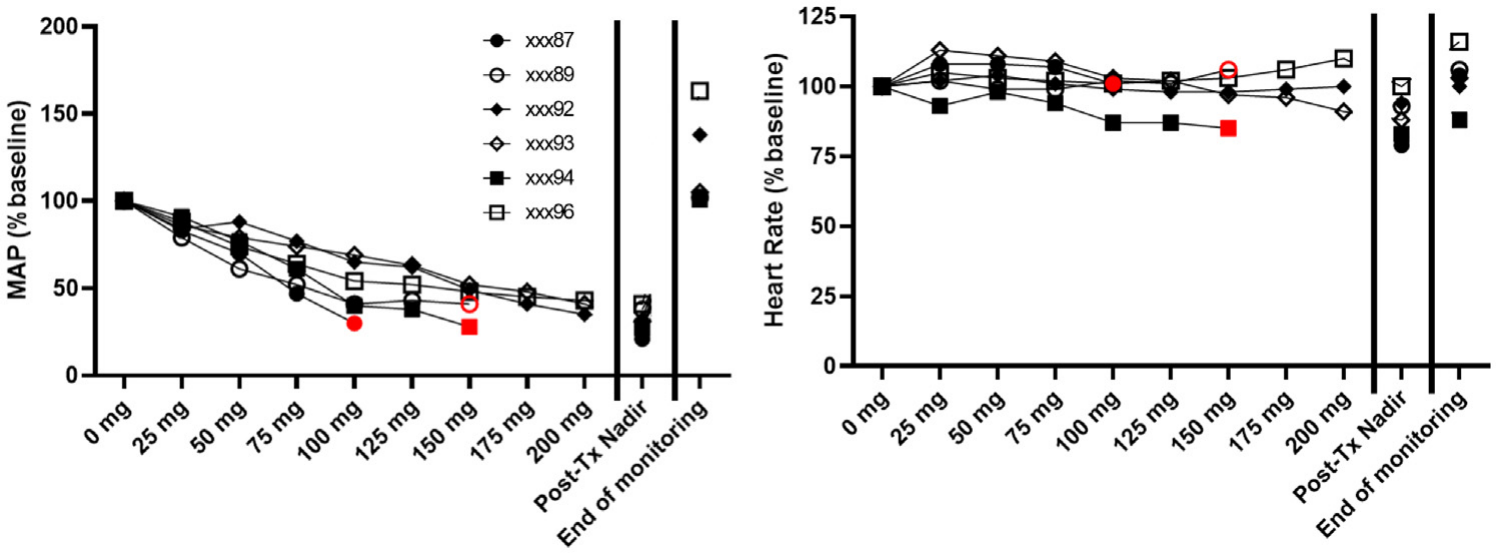
Intravenous (IV) administration of polidocanol solution (PS) in baboons results in rapid and dose-related effects on blood pressure (BP). Female baboons (n = 6) underwent placement of IV catheters and arterial lines under general anesthesia. After establishing baseline assessments, we used a syringe pump to administer 1% PS (pharmaceutical grade) at a rate of 1 mL/min (10 mg/min) up to 200 mg (20 mL), with continuous measurement of cardiovascular parameters through an arterial line throughout treatment and for 15 to 30 minutes after stopping infusion. A 12-lead electrocardiogram (ECG) was completed every 1 to 2 minutes. (Left) Mean arterial pressure (*MAP*). (Right) Heart rate (HR) as a percent of baseline. Only three of the animals tolerated the entire target dose of 200 mg PS. Treatment was terminated prematurely when the BP decreased by more than 30% from baseline (red symbols). No clinically important (±30%) change in HR occurred in any of the animals. Post-Tx nadir indicates lowest result record for each parameter. *Tx*, treatment.

Having determined that doxycycline did not contribute to the cardiovascular effects in rats, we performed additional experiments with the IV administration of polidocanol only in baboons. Similar to rats, the IV administration of PS in baboons resulted in a rapid and dose-related effects on BP, HR, EtCO_2_ but not O_2_ sat (Fig 6). All of the animals experienced a more than 50% decrease in the pulse pressure (range, 52%-92%). The effects reversed after the discontinuation of treatment in all six females.

**Fig 7:**
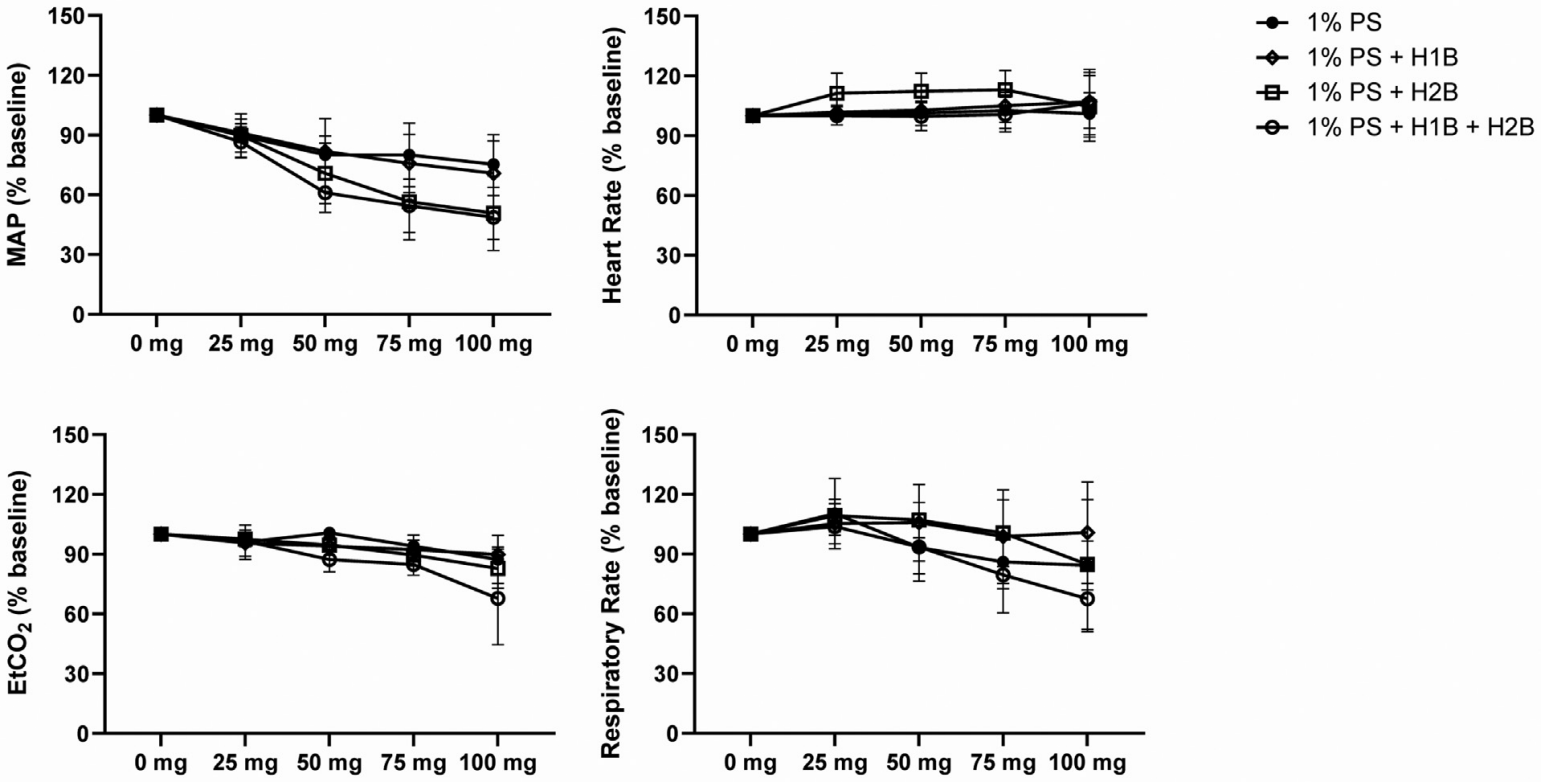
Antihistamine treatment does not mitigate the adverse cardiovascular effects of intravenous (IV) polidocanol solution (*PS*). Baboons were pretreated with diphenhydramine (*H1B*), ranitidine (*H2B*), or both (H1B + H2B), or received no pretreatment before IV administration of 1% PS (1 mL/min, target dose 10 mL = 100 mg). Administration of PS resulted in a rapid dose-dependent decrease in blood pressure (BP) and respiratory rate, neither of which was prevented by antihistamine pretreatment. Data (normalized to baseline) presented as mean ± standard deviation (normalized to percent baseline). n = 3-6 per group. *EtCO*_*2*_, end-tidal CO_2_.

To test the hypothesis that histamine release mediated the adverse cardiovascular effects of polidocanol, we pretreated anesthetized baboons with diphenhydramine (H1B), ranitidine (H2B), both diphenhydramine and ranitidine (H1B+H2B), or no additional treatment (C) before the administration of 1% PS through a peripheral IV catheter via an infusion pump. All of the antihistamine-treated females also received pretreatment with cromolyn sodium 20 mg by nebulizer. Similar to our other experiments with IV PS and pressurized TC PF, we found a dose-related decrease in the BP. Although this change was not associated with a decrease in the HR in most animals, we observed a decrease in the ETCO_2_ suggestive of decreased perfusion. Neither treatment with H1B or H2B prevented these adverse effects, with broad overlap between the treatment groups. Notably, the most severe changes occurred with combined H1B+H2B (Fig 7).

### IP administration of PF does not result in adverse hemodynamic effects

During TC delivery, polidocanol could enter the vasculature through endometrial blood vessels and lymphatics or as a result of peritoneal absorption after spilling through the fallopian tubes. To further evaluate our hypothesis that adverse cardiovascular effects result from endometrial uptake of polidocanol during TC administration of pressurized PF, and not from peritoneal uptake, we performed direct IP administration in rats and baboons.

**Fig 8:**
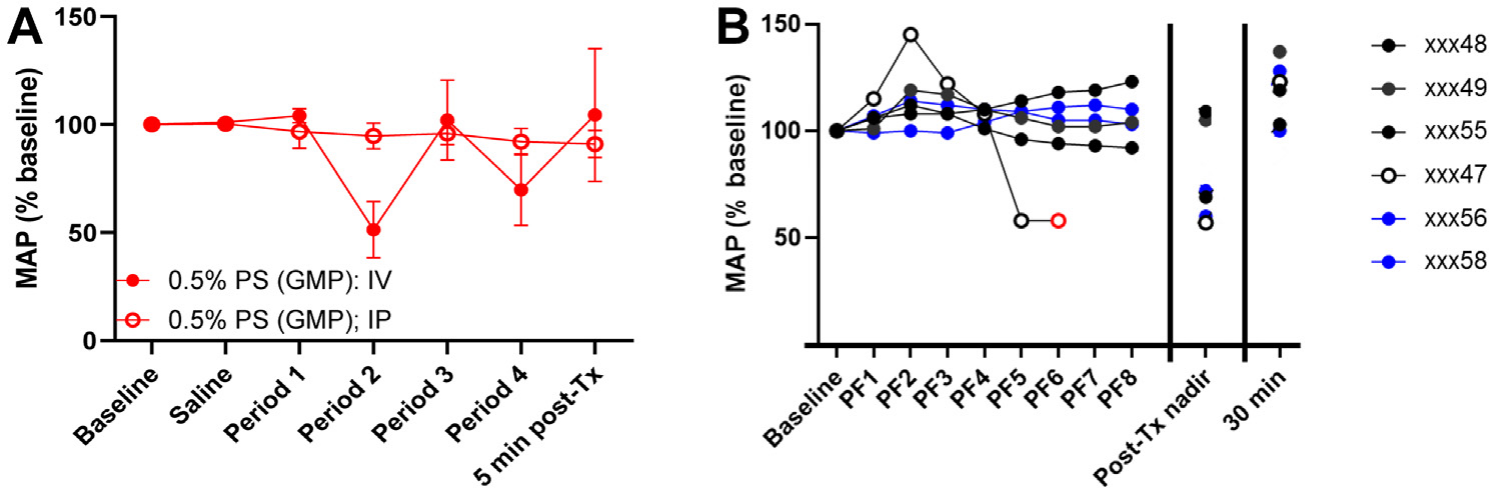
Intraperitoneal (IP) administration of polidocanol foam (PF) does not result in adverse blood pressure (BP) effects. **(A)** Rats (n = 6) received 0.5% PS intravenously (IV) or IP. IV administration resulted in a reversible decrease in the mean arterial pressure (*MAP*), an effect not seen with IP administration. Data represent the start of each period: saline for 3 minutes (0.3 mL); period 1, 0.5% PS for 5 minutes (0.5 mL); period 2, no infusion (3-7 minutes); period 3, 0.5% PS for 2 minutes (0.2 mL); and period 4, recovery (5-20 minutes). Data are presented as mean ± standard deviation (normalized to percent baseline). **(B)** Baboons received up to 40 mL of 5% foam following laparoscopy (black circles, n = 4) or through a direct IP injection (blue circles, n = 2). PF1-8 indicate successive 5-mL (50-mg) aliquots of PF. Post-treatment (*Tx*) nadir reflects the lowest MAP reading after Tx and 30 minutes, the reading 30 minutes after completion of the infusion. Data reflects start of each period normalized to baseline. Red symbol indicates premature discontinuation PF infusion in one female (laparoscopic group, open black circle) owing to a more than 30% decrease in the MAP; recovery was prompt.

Rats that received a 0.1-mL/min IV infusion of 0.5% PS developed an approximately 50% decrease in BP from baseline, whereas animals that received the same treatment and dose IP showed no change in BP (*P* < .05) (Fig 8, *A*).

Four baboons received IP administration of 5% PF (40 mL, 400 mg) at the time of laparoscopy, and two additional females through a direct IP injection. Three of the four females treated at the time of laparoscopy tolerated the target 40-mL 5% PF dose without clinically important changes in MAP (Fig 8, *B*, black closed circles). One female received only 30 mL of PF after reaching the stopping point of a 30% decrease (Fig 8, *B*, black open circles). Notably, the administration of foam was observed subjectively to require considerable force in this female not perceived in the other three, suggesting embedment of the catheter tip in the peritoneal surface, resulting in pressurization. Moreover, the decrease in MAP was transient with recovery beginning within 5 minutes of discontinuation and complete at 30 minutes. The two animals that received direct IP injections showed no clinically important changes in BP (Fig 8, *B*, blue circles).

### Adverse cardiovascular effects of pressurized TC administration of PF in baboon

We simultaneously performed transthoracic echocardiography during pressurized TC administration of up to 40 mL of 5% PF delivered with a Cook HSG catheter with the balloon inflated on four additional female baboons. We selected these females because we had noted significant vascular uptake of contrast agent and tubal occlusion during prior HSG evaluations, but two animals (xxx43, xxx49) demonstrated tubal patency during the echo procedures, with one of these (xxx49) showing no apparent vascular uptake. All but one animal (xxx91) received pretreatment with diphenhydramine 25 mg IV and cromolyn sodium 20 mg by nebulizer before the polidocanol infusion. For all echo measurements, animal xxx49, the female with tubal patency and no evidence of significant vascular uptake, tended to have less adverse effects on LV function than the other animals. We did not see any evidence of an effect of antihistamine treatment.

**Fig 9:**
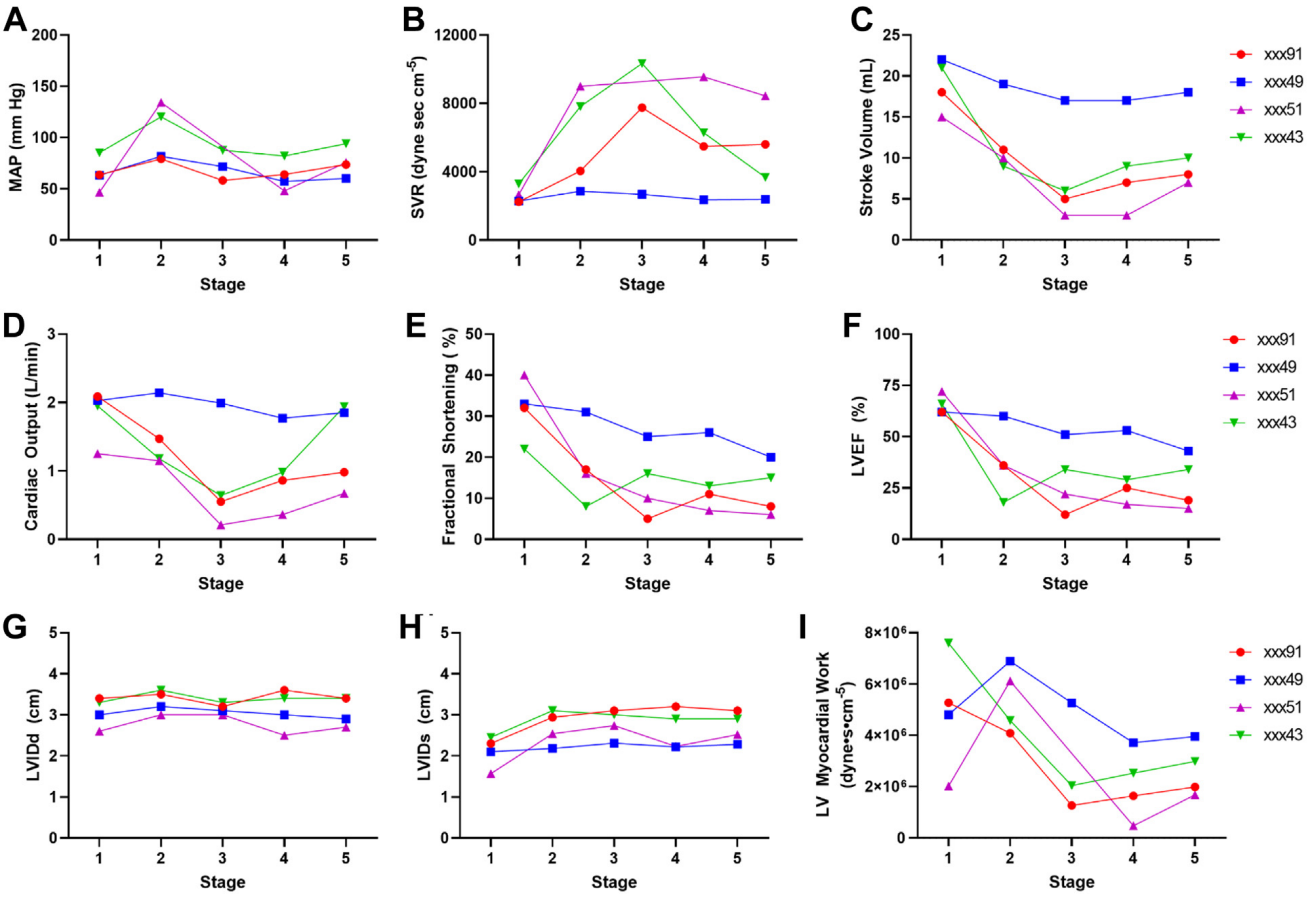
Transthoracic echocardiography during pressurized transcervical (TC) administration of 5% polidocanol foam (PF) revealed LV dysfunction. Mean arterial pressure **(A)**; systemic vascular resisitance **(B)**; stroke volume **(C)**; cardiac output **(D)**; fractional shortening **(E)**; left ventricular ejection fraction **(F)**; left ventricular end diastolic dimension **(G)**; left ventricular end-systolic dimension **(H)**; and left ventricular myocardial work **(I)**. Two animals (xxx91, xxx51) demonstrated prominent vascular uptake of contrast agent and tuba occlusion during hysterosalpingogram (HSG), one (xxx43) prominent vascular uptake and tubal patency, and one (xxx49) tubal patency with no apparent vascular uptake. For all echo measurements, animal xxx49, the female with tubal patency and no evidence of significant vascular uptake, tended to have less adverse effects on LV function than the other animals. *LV*, left ventricular; *LVEF*, left ventricular ejection fraction; *LVIDd*, LV end-diastolic dimension; *LVIDs*, LV end-systolic dimension; *MAP*, mean arterial pressure; *SVR*, systemic vascular resistance.

Arterial pressure (MAP) tended to increase early during the PF infusion but then returned to baseline values, whereas SVR, which takes into account cardiac output, increased markedly during PF infusion and remained high in all three animals that demonstrated vascular uptake during the TC infusion (Fig 9, *A* and B). Stroke volume, whether measured by LV outflow tract flow or by Simpson’s biplane, decreased early during PF injection in the three animals with vascular uptake, and remained low, resulting in a decreased cardiac output that persisted long after the injection was completed (Fig 9, *C* and D). Other metrics of LV function such as fractional shortening and LV ejection fraction showed similar patterns with a severe decrease in affected animals, which did not fully recover by the final study (Fig 9, *E* and F). Decreased function was associated with an increased in LV end-systolic dimension without evidence for acute dilation by end-diastolic dimension (Fig 9, *G* and H). LV myocardial work, an index that takes into account afterload, initially increased in several animals during PF infusion, but eventually decreased to values below baseline in all animals that had evidence of decreased LV function (Fig 9, I). These findings imply that decrease in LV function early during PF infusion may be influenced by load but progressive worsening occurs owing to a direct decrease in myocardial performance. We observed no major changes in LV diastolic measurements, including early diastolic velocity of the mitral annulus (eʹ) nor estimated LV filling pressure by the ratio of transmitral E/eʹ (data not shown).

**Fig 10:**
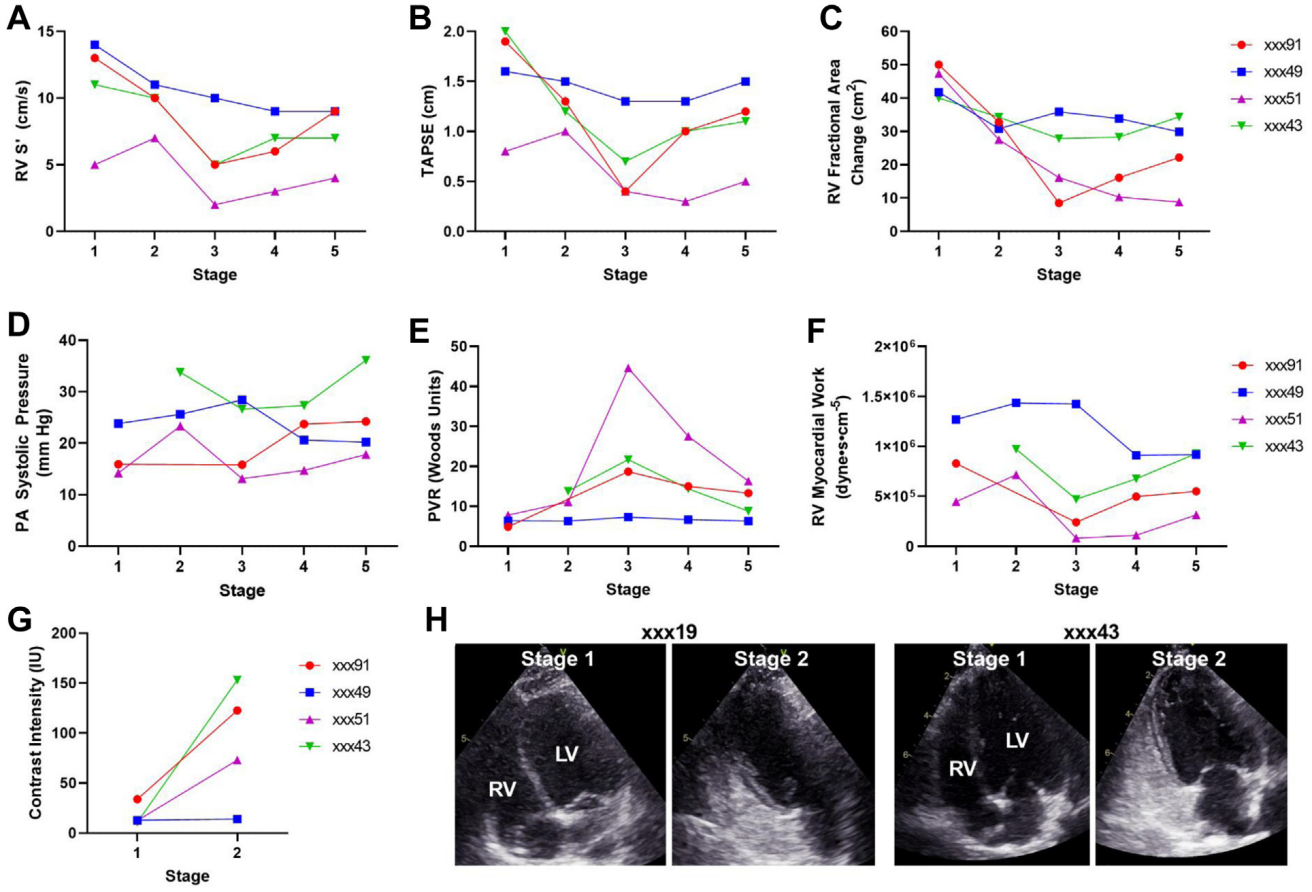
Transthoracic echocardiography during pressurized transcervical (TC) administration of 5% polidocanol foam (PF) resulted in a similar temporal pattern of right ventricular (*RV*) dysfunction as seen for the left ventricle (*LV*). A progressive decrease in the RV peak systolic velocity of the lateral tricuspid annulus (*Sʹ*), tricuspid annular plane excursion (*TAPSE*), and RV fractional area change were seen during active PF infusion **(A-C)**. PVR increased more than pulmonary artery (*PA*) systolic pressure secondary to changes in stroke volume **(D, E)**. RV myocardial work remained either unchanged or reduced implying again a mixed effect **(F)**. Contrast enhancement in the form of cavitation nuclei, suggestive of PF bubbles or droplets, was observed only after initiating PF injection and were seen only in the right heart chambers with no transition of contrast enhancement to the left sided chambers **(G, H)**. Contrast enhancement was not observed in animal xxx49, the animal noted to have less severe hemodynamic and functional deficits. *PVR*, pulmonary vascular resistance.

We found a similar temporal pattern of right ventricular (RV) dysfunction as seen for the LV. A progressive decrease in RV peak systolic velocity of the lateral tricuspid annulus (Sʹ), tricuspid annular plane excursion, and RV fractional area change were seen during active PF infusion (Fig 10, A-*C*). Similar to what was found for the systemic circulation, PVR increased more than pulmonary artery systolic pressure secondary to changes in stroke volume (Fig 10, *D* and E). Again, animal XXX49 tended to have less severe hemodynamic and functional deficits than other animals. RV myocardial work remained either unchanged or decreased, implying again a mixed effect of load-related decrease in function early and impaired myocardial performance late (Fig 10, *F*).

Contrast enhancement in the form of cavitation nuclei, suggestive of PF bubbles or droplets, were observed only after initiating PF injection and were seen only in the right heart chambers with no transition of contrast enhancement to the left sided chambers (Fig 10, *G* and H). Contrast enhancement was not observed in animal xxx49.

## Discussion

PS and foam are approved agents for venous sclerosis, and are used off-label for a variety of indications. Our group has reported that TC delivery of PF will result in de-epithelialization of the intramural portion of the fallopian tube in nonhuman primates and formation of a permanent collagen scar.^23^ We have performed a proof-of-concept contraception study in baboons that demonstrated prevention of pregnancy in 80% of the females that received a single treatment with 5% PF delivered using a standard HSG catheter.^15^ However, TC administration of foam results in considerable pressurization of the endometrial cavity as the small fallopian tube diameter of the baboon results in high resistance to flow. This pressurization results in the uptake of fluids and foam into the extensive vascular plexus of the endometrium. Vascular uptake of fluids is a known complication of hysteroscopic surgery in women, with deaths reported.^24^ The considerable vascular remodeling that occurs in the endometrium during the menstrual cycle results in a loss of microvascular endothelial integrity that contributes to this process, but pressurization plays a critical role.^25^ Our data suggest that, during TC PF procedures in baboons, the vascular uptake of polidocanol that occurs as a result of uterine cavity pressurization is the main contributor to cardiovascular toxicity.

Because TC delivery of PF represents a new route of delivery, we conducted toxicology studies to evaluate safety of the approach to support future clinical trials of PF for permanent contraception. We found significant dose-dependent adverse cardiovascular effects with pressurized TC administration of 5% PF and PDF, an alternative formulation. Because the 5.0% concentration of polidocanol is considerably higher than the 0.5% and 1.0% formulations currently approved for clinical use for venous sclerosis, and we do not have a complete understanding of the pharmacokinetics of polidocanol uptake with intrauterine administration, we conducted controlled experiments of dose and rate using IV infusion. Our results in rats confirm that adverse hemodynamic effects occur rapidly during IV administration, and that this effect is not related to or increased by the addition of doxycycline. The direct IV administration of PS in baboons produces the same response. In contrast, TC administration of PF without inflation of the balloon, which permits excess foam to drain through the cervix without pressurization, or direct IP administration of PF do not result in severe adverse hemodynamic effects. These experiments support the premise that pressurization within the uterine cavity results in endometrial vascular uptake of PF that generates the observed effects. To mitigate the adverse effects and improve safety and efficacy, a selective catheter system to deliver foam to the fallopian tubes without cavity pressurization is under development to decrease endometrial vascular uptake.

Pressurization of the uterine cavity occurs during many gynecologic surgical (eg, hysteroscopy) and imaging (eg, HSG) procedures and is not associated with adverse cardiovascular effects. All of the baboons used in our experiments underwent a baseline HSG procedure before administration of PF; we did not observe adverse HR and BP changes during these examinations (data not shown).

Although allergic responses after polidocanol administration in humans have been reported, the transient and dose-related hemodynamic features we observed in animals not previously exposed to polidocanol are not consistent with a type I or II hypersensitivity reaction. To explore the hypothesis of a potential CARPA effect, we pretreated baboons with histamine receptor blockers before IV PS. Our results did not support a benefit of HR1 or HR2 blockade. Rather, antihistamine treatment tended to result in more severe adverse changes.

We performed echocardiography to provide insights into the cardiovascular toxicity of polidocanol. The integration of echocardiographic and hemodynamic data from these experiments is complex. We can conclude unequivocally that gaseous or emulsion-based bodies, likely containing polidocanol, are partitioning in a pressure-dependent fashion into the venous circulation. There is no transition to the left-sided chambers, indicating either that these particles are sufficiently large enough to lodge in the pulmonary circulation, are taken up by other mechanisms (eg, pulmonary macrophages or Cʹ-mediated binding), or lose their gas volume during pulmonary transit. The absence of left-sided contrast does not exclude the transit of polidocanol to the systemic circulation, only that any transit is not in the form of a large gas-containing particle that can be detected by ultrasound techniques designed to receive cavitation energy. LV and RV dysfunction with subsequent decrease in cardiac output are seen very early in the administration. Based on the changes in PVR and SVR, and the temporal changes in RV and LV myocardial work, it is likely that afterload mismatch played a role in the decrease in ventricular function early during the infusions. In particular, the RV is less tolerant of acute increases in afterload and the degree of PVR that we observed was severe (>15 Woods units) in animals that also had severe decrease in ventricular function. However, direct effects on ventricular function are very likely, particularly late in the infusion, based on the severe decrease in LV function at a time when SVR was increased but systemic BP, based on myocardial work values, was not. The relative sparing from adverse effects in the animal lacking evidence for contrast enhancement provides a direct link between blood pool absorption of polidocanol and adverse cardiovascular effects. It is unlikely that physical obstruction of the pulmonary microcirculation from large gas bubbles was the primary cause of cardiovascular changes. Massive microvascular obstruction would have likely resulted in decrease in LV dimensions, severe septal shift, and a decrease in E/eʹ, which were not seen.

The changes in SVR are complex. It is possible that PD-related increases in SVR contributed to impairment in ventricular performance. However, SVR is also known to increase in response to an acute decrease in cardiac output to maintain perfusion pressure in vital organs. The finding of moderate to severe LV dysfunction in the presence of high SVR but relatively normal SBP implies that direct changes to LV function were dominant mechanisms for the high SVR. During clinical administration of polidocanol, an increase in BP and HR may indicate the potential for rapid deterioration and prompt termination of treatment. Alternatively, echocardiographic monitoring for systemic absorption can also be considered as a potential safety measure.

Thus, our experimental results demonstrate that polidocanol infusion results in adverse cardiovascular changes owing to vascular and probably direct myocardial toxicity. Currently, we do not understand the cellular mechanism of this toxicity, but it seems to be dose related, transient, and reversible. Palmitoyl carnitine, a chemically similar long-chain fatty alcohol surfactant has been shown to irreversibly inhibit Na^+^-K^+^-ATPase in canine cardiac myocyte sarcolemma.^26^ Oexle et al^27^ reported that polidocanol perfusion of the guinea pig heart caused an irreversible decrease in frequency and delay of atrioventricular and intraventricular conduction consistent with blockade of fast sodium channels. Our results from ECG monitoring during polidocanol treatment of baboons do not support abnormality of the conduction system; we observed sinus rhythm even during periods of profound hypoperfusion. However, the decrease in BP with a decrease, rather than compensatory increase, in the HR in rats suggests a conduction abnormality may occur in rodents, consistent with the effects observed by Oexle et al. We did not conduct echocardiogram studies in rats.

Cardiac arrest and myocardial infarction after PF sclerotherapy have been reported.^18^^,^^28^^,^^29^ A major weakness of our study is that we did not obtain troponin or other enzymes to evaluate myocardial damage and we do not have polidocanol blood concentrations. We did not observe any residual clinical signs of behavioral features (eg, decreased activity, pain, poor feeding) after recovery suggestive of permanent cardiovascular compromise among any of the treated animals, even those with significantly prolonged treatment-related hypotension. Furthermore, we conducted complete necropsy evaluations in all of the animals reported in this study, which revealed no abnormal gross or histologic abnormalities in the heart.

We did not evaluate the potential impact of sex differences, because we used female animals for all of our experiments. Further experiments are needed to confirm whether the cardiovascular effects of intravascular administration of polidocanol are generalizable to male animals.

Polidocanol enters the systemic circulation during TC intrauterine administration with pressurization. Although pressurization does not normally occur with administration of PF or PS during administration with approved routes of IV administration, it may occur with some off-label uses. For example, Gupta et al^9^ reported a case of life-threatening cardiac collapse in a 3-year-old boy treated with 10 mL of 3% PS for an aneurysmal bone cyst of the femur. The child developed tachycardia and a decrease in BP from 96/52 mm Hg to 80/30 mm Hg after injection of the initial 3 mL. The clinicians waited 15 minutes for the BP to stabilize, and then continued the infusion. This resulted in a BP decrease from 100/70 mm Hg to 50/20 mm Hg and ventricular tachycardia, findings similar to those observed during pressurized TC PF administration and IV PS in baboons. The child was treated with amiodarone and supportive care, and stabilized within 20 to 30 minutes.

In summary, our experiments demonstrate that hypotension during polidocanol treatment occurs as a consequence of low cardiac output secondary to impaired biventricular function. The onset is acute, dose dependent and precipitous, and resolves slowly upon discontinuation of the polidocanol infusion with appropriate supportive care. The physiologic features of this response are not explained by classical allergy or by CARPA. We propose that many of the adverse events reported following therapeutic use of PS and PF occur owing to this direct effect on both the pulmonary vasculature and the myocardium, and not as an allergic mechanism. Further investigations of this response mechanism may provide insight into potential therapeutic interventions to improve safety of polidocanol treatment.

## Author Contributions

Conception and design: JJ, OS, JL

Analysis and interpretation: JJ, PL, JG, JL

Data collection: JJ, PL, MR, JG, SY, EM, TG, JH, JL

Writing the article: JJ

Critical revision of the article: JJ, PL, MR, JG, SY, EM, TG, JH, OS, JL

Final approval of the article: JJ, PL, MR, JG, SY, EM, TG, JH, OS, JL

Statistical analysis: JJ

Obtained funding: JJ

Overall responsibility: JJ

## Figures and Tables

**Supplementary Table I:** Mean arterial pressure (*MAP*) in baboons in response to transcervical pressurized administration of polidocanol/doxycycline foam (PDF), effect of dose

MAP (% baseline)	Target dose = 200 mg polidocanol				Target dose = 400 mg polidocanol				
	xxx85	xxx88	xxx89	xxx90	xxx87	xxx92	xxx93	xxx94	xxx96
Delivered dose (mg)	200	200	200	200	320	400	300	200	310
0	100	100	100	100	100	100	100	100	100
25	100	119	100	166	229	96	100	100	99
50	105^a^	124	147	171	227	185	b	98	b
75	110^a^	141	164	191	215	179	174	155	182
100	120^a^	130	177	169	185	181	122	147	199
125	145^a^	127	151	143	169	153	109	120	197
150	170^a^	106	130	97	144	158	89	100	179
175	195^a^	122	85	81	158	140	93	73	161
200	207	95	81	0^b^	140	140	43	0^b^	148
225	–	–	–	–	137	132	0^b^	c	154
250	–	–	–	–	112	125	0^b^	c	125
275	–	–	–	–	94	113	0^b^	c	110
300	–	–	–	–	98	113	0^b^	c	90
325	–	–	–	–	77	106	c	c	0^b^
350	–	–	–	–	c	111	c	c	c
375	–	–	–	–	c	96	c	c	c
400	–	–	–	–	c	96	c	c	c
Post-treatment nadir	117	59	64	41	67	94	0	0	42
Post-treatment 35 min	133	111	77	136	110	111	102	165	104

Female baboons received PDF: target dose 200 mg polidocanol ( n = 4); target dose 400 mg polidocanol (n = 5). Although all four animals in the 200-mg group tolerated the full target dose, only one of five in the 400-mg group tolerated the full target dose, and two animals in this group required intervention (xxx93 received two doses of epinephrine while xxx94 received one dose). Data are presented as percent baseline.

a Owing to the infusion pump being set slightly faster than the target rate, some data points had to be extrapolated (xxx85 still received the target dose of 200 mg).

b Data missing owing to a loss of signal from monitoring equipment or blood pressure was presumed "0" if it became nonpalpable.

c Administration of PDF stopped before reaching the target dose.

**Supplementary Table II:** Heart rate in baboons in response to transcervical pressurized administration of polidocanol/doxycycline foam (*PDF*)

Heart rate (% baseline)	Target dose = 200 mg polidocanol				Target dose = 400 mg polidocanol				
	xxx85	xxx88	xxx89	xxx90	xxx87	xxx92	xxx93	xxx94	xxx96
Delivered dose (mg)	200	200	200	200	320	400	300	200	310
0	100	100	100	100	100	100	100	100	100
25	99	103	106	102	109	98	104	97	101
50	111^a^	114	123	108	113	129	133	111	107
75	110^a^	114	107	107	112	129	140	112	110
100	112^a^	118	110	104	111	130	137	113	111
125	117^a^	115	110	103	110	124	140	116	109
150	121^a^	114	109	102	109	128	138	117	107
175	122^a^	110	112	97	109	126	129	115	111
200	121	111	107	96	111	127	116	106	111
225	–	–	–	–	110	124	b	c	113
250	–	–	–	–	109	122	109	c	118
275	–	–	–	–	109	119	113	c	119
300	–	–	–	–	109	119	c	c	114
325	–	–	–	–	106	117	c	c	c
350	–	–	–	–	c	117	c	c	c
375	–	–	–	–	c	114	c	c	c
400	–	–	–	–	c	114	c	c	c
Post-treatment nadir	100	91	98	85	99	105	101	97	95
Post-treatment 35 min	103	91	98	88	99	108	132	99	95

Female baboons were administered PDF with four given a target dose of 200 mg polidocanol and five given a target dose of 400 mg polidocanol. All animals in the 200-mg group tolerated the full target dose. Only one of the five animals in the 400-mg group tolerated the full target dose, and two received epinephrine (xxx93, xxx94). Heart rate was not dramatically affected by PDF infusion. Data are presented as percent baseline.

a Owing to the infusion pump being set slightly faster than the target rate, some data points had to be extrapolated (xxx85 still received the target dose of 200 mg).

b Data missing owing to loss of signal from monitoring equipment.

c Administration of PDF stopped before reaching the target dose.

**Supplementary Table III:** Female baboons were administered polidocanol/doxycycline foam (*PDF*) with four given a target dose of 200 mg polidocanol and five given a target dose of 400 mg polidocanol

O_2_ sat (% baseline)	Target dose = 200 mg polidocanol				Target dose = 400 mg polidocanol				
	xxx85	xxx88	xxx89	xxx90	xxx87	xxx92	xxx93	xxx94	xxx96
Delivered dose (mg)	200	200	200	200	320	400	300	200	310
0	100	100	100	100	100	100	100	100	100
25	100	100	100	100	100	100	100	100	100
50	100^a^	100	100	100	100	100	100	101	100
75	100^a^	100	100	100	100	100	100	101	100
100	100^a^	100	100	100	100	99	100	101	100
125	100^a^	100	100	99	100	99	100	101	100
150	100^a^	93	100	99	100	100	100	101	100
175	100^a^	b	100	99	100	99	100	100	100
200	100	b	100	99	100	100	100	89	100
225	–	–	–	–	100	100	b	c	100
250	–	–	–	–	100	100	b	c	100
275	–	–	–	–	100	100	b	c	100
300	–	–	–	–	100	100	b	c	100
325	–	–	–	–	100	100	c	c	c
350	–	–	–	–	c	100	c	c	c
375	–	–	–	–	c	100	c	c	c
400	–	–	–	–	c	100	c	c	c
Post-treatment nadir	100	99	98	98	100	100	100	89	100
Post-treatment 35 min	100	99	99	98	100	100	100	99	100

All animals in the 200 mg group tolerated the full target dose. Only one of the five animals in the 400-mg group tolerated the full target dose, and two animals in this group required intervention (xxx93 received two doses of epinephrine while xxx94 received one dose). Data are presented as percent baseline.

a Owing to the infusion pump being set slightly faster than the target rate, some data points had to be extrapolated (xxx85 still received the target dose of 200 mg).

b Data missing owing to loss of signal from monitoring equipment.

c Administration of PDF stopped before reaching target dose.

**Supplementary Table IV:** Nonpressurized transcervical delivery of polidocanol foam (*PF*) and pressurized transcervical delivery of PF

	xxx16	xxx17	xxx19	xxx20	xxx22
Nonpressurized cervical delivery					
Polidocanol (mg)					
Target	400	400	400	400	400
Delivered	400	400	400	400	400
Mean intrauterine pressure during PF infusion (mm Hg)	Missing^a^	54	51	38	99
>30% BP decrease?	Y	Y	N	N	N
Polidocanol (mg) at 30% decrease	350^b^	400	n/a	n/a	n/a
Resolution	Rapid	Rapid	n/a	n/a	n/a
Pressurized transcervical delivery					
Polidocanol (mg)					
Target	400	400	400	400	400
Delivered	320	100	150	400	400
Mean intrauterine pressure during PF infusion (mm Hg)	692	103	196	265	153
>30% BP decrease?	Y	Y	Y	Y	N
Polidocanol (mg) at 30% decrease	320	100	150	350	n/a
Resolution	Slow	Slow	n/a	Medium	n/a

*BP*, Blood pressure; *N*, no; *Y*, yes.

To administer PF without pressure, a Cook HSG catheter was inserted transcervically but without the balloon inflated. Two of five animals demonstrated a greater than 30% decrease from baseline in the mean arterial pressure (MAP), although both occurred near or following the completion of PF infusion and resolved rapidly without intervention.

To administer PF under pressure, a Cook HSG catheter was inserted transcervically with the balloon inflated. We observed a greater than 30% decrease in MAP from baseline during or after PF delivery in all five animals, with three animals requiring premature termination of PF infusion and experiencing a slow recovery from the induced hypotension.

a Intrauterine pressure was not recorded for this animal.

b We observed a 30% decrease in MAP after approximately 350 mg polidocanol was administered although she tolerated the entire target dose of 400 mg polidocanol.
