# Supplementary material for: Systemic polidocanol from intravenous or pressurized intrauterine administration produces reversible cardiovascular toxicity
Source: JVS Vasc Sci. 2022 Oct 20;3:316–35. doi: 10.1016/j.jvssci.2022.08.002 (PMC9692028; doi:10.1016/j.jvssci.2022.08.002)
Supplement: Supplementary material [file mmc1.pdf]

Supplemental Information on Nonhuman primate protocols

Institutional Animal Care and Use Committees (IACUC) at the Oregon National Primate Research Center (ONPRC) and Kenya Institute of Primate Research approved all of the baboon procedures at their respective institutions. Animal husbandry provided by both institutions is in accord with the National Institutes of Health (NIH) Guidelines for Care and Use of Laboratory Animals [1], with all animals maintained in social housing groups. We conducted all of the surgical procedures under general anesthesia using IACUC-approved protocols. Adult female baboons (Papio anubis, Papio hamadryus) Age: 12±7 years; weight 17±8 kg) used in the study underwent sedation with ketamine (10 mg/kg IM, Putney, Dublin, OH) before endotracheal intubation and induction and maintenance of general anesthesia with isoflurane (WI, Boise, ID) inhalation.

Housing: All of the baboons at both institutions were housed socially in groups of 8-12 females. At ONPRC, the females were co-housed with a single (vasectomized) male. At Kenya IPR, we co-housed groups of 8 females with a fertile male. At both sites, animals had free access to both indoor and outdoor areas. The housing space at both centers included structures for exploration and play that also provided an opportunity for climbing and perches for sitting, and space for grooming activity. We included a variety of foraging devices and manipulada for enrichment. Prior to scheduled procedures, females were temporarily housed in cages that exceeded European Union standards for space. In all cases, females had a minimum of grooming access to at least one other female at all times, including when caged. Animal care technician cleaned housing and cage areas on a daily basis.

Diet: Baboons at both sites received a standard diet of Purina Monkey Chow biscuits with no restrictions provided daily. In addition, they received supplements of fresh fruits and vegetables on a daily basis, and had unrestricted access to water. In keeping with standard pre-operative precautions, we withheld water and/or food temporarily (e.g. 6-12 hrs) from animals undergoing procedures that required anesthesia.

The following pages provide additional details on the approved procedures used for pre- and post-operative care and for humane euthanasia (not an outcome reported in this manuscript). The procedures for anesthesia used at Kenya IPR did not differ from those presented for ONPRC.

1. Guide for the Care and Use of Laboratory Animals: Eighth Edition: The National Academies Press; 2011.

Institutional Animal Care and Use Program	
Type: Standardized Non-Surgical Procedure	Date Effective: 04/16/2019
Species: Anubis Baboon, Baboons, Cynomolgus Macaque, Hamadryas Baboon, Japanese Macaque, Macaque Spp., Rhesus Macaque, Southern Pig-tailed Macaque, Vervet Monkey	
Standard Pre-operative Care Procedure	

1. **Fasting prior to surgery**
   Food will typically be withheld for approximately 12 hours prior to the procedure. Water will not be withheld.

2. **Prophylactic antibiotics used during preoperative care**
   Antibiotics are not standard for this procedure, but cefazolin (at 25 mg/kg IV or IM) or enrofloxacin (at 10 mg/kg IM) may be given prior to the procedure at the discretion of the surgical veterinarians.

3. **Pre-operative analgesics**
   Local or systemic analgesia may be given prior to and/or following the procedure at the discretion of the veterinarians.

1. **Anesthesia agent(s) including the name, dose, route, frequency of administration and duration.**

   Animals are sedated 8-20 mg/kg ketamine administered intramuscularly then intubated with an appropriately-sized endotracheal tube and general anesthesia is induced with ~3% isoflurane until the patient begins to lose reflexes and heart rate begins to slow. Inhalant anesthesia is maintained @ 1-2% isoflurane which may be adjusted based on the physiologic parameters of the patient. Inhalant anesthetics are combined with 100% oxygen, room air, or a combination of oxygen and room air administered at a rate of 1-1.5 L/min.

2. **Criteria for assessing adequacy of anesthesia.**
   The following criteria may be used to assess adequacy of anesthesia and animal intraoperative well-being during the procedure:

   - Body temperature via esophageal or rectal temperature probe
   - Heart rate and pulse character via palpation, auscultation, pulse oximetry and electrocardiography
   - Blood pressure via indirect blood pressure cuff and/or direct percutaneous arterial line
   - Peripheral oxygen saturation via pulse oximetry
   - Respiratory rate and pattern
   - End tidal Carbon Dioxide
   - Capillary refill time
   - Absence of palpebral response to touching the medial canthus
   - Jaw tone
   - Color of mucous membranes at gums or conjunctiva

1. Refer to GL-57 for all approved injectable anesthetics.

   Options include the below:

   a. 3-20 mg/kg ketamine administered intramuscularly;
      - 3-5 mg/kg is recommended for increasing compliance with procedures for which the animal does not require removal from the home cage.
      - 7-10 mg/kg is standard dosing for most 15-30 minute procedures.
      - 20 mg/kg is typically reserved for pre-necropsy sedation.

   b. 0.1- 0.2 mg/kg midazolam IM may be administered intramuscularly either in the same syringe with ketamine or following ketamine administration.

   c. 3-5 mg/kg Telazol (tiletamine-zolazepam) administered intramuscularly;
      - Animals requiring additional sedation following the initial telazol injection should be administered a small dose (3-5 mg/kg) of ketamine (IV or IM) rather than an additional dose of Telazol unless otherwise directed by a veterinarian.
      - Telazol may be partially reversed (zolazepam component) to reduce respiratory depression and/or hasten recovery with 0.01 mg/kg flumazenil intravenously; reversal doses may be repeated up to 2 times based on consultation with veterinary staff;

   d. 3-20 mg/kg ketamine with 5-25 µg/kg dexmedetomidine administered intramuscularly.
      - Dosing of dexmedetomidine should correlate with the needed depth and length of anesthesia, with brief, non-painful procedures dosed at the low end and vice versa.
      - 25 µg/kg doses are typically reserved for pre-necropsy sedation where procedures are required prior to necropsy.
      - Total dose of dexmedetomidine must not exceed 150 µg (0.3ml).
      - Dexmedetomidine may be reversed with a volume of atipamezole equal to the volume of the dexmedetomidine administered to reduce cardiovascular effects (bradycardia, hypotension) and hasten recovery.

- Atipamezole is administered intramuscularly, and may be repeated up to 2 times based on consultation with veterinary staff.

e. Fasting for anesthetic procedures, as well as monitoring during and recovery from anesthesia are performed as outlined in GL-057, GL-105 and GL-075. Any deviations from these SOPs must be clearly outlined within the IACUC protocol.

Potential Adverse Outcomes

While uncommon, hypothermia, hypoglycemia, respiratory insufficiency, respiratory distress or arrest, and cardiac insufficiency or arrest leading to severe neurologic or organ compromise or death are potential sequelae to anesthesia. All appropriate measures will be taken to avoid these potential adverse sequelae.

Institutional Animal Care and Use Program	
Type: Standardized Surgical Preparation Procedure	Date Effective: 10.16.2018
Species: Anubis Baboon, Baboons, Cynomolgus Macaque, Hamadryas Baboon, Japanese Macaque, Macaque Spp., Rhesus Macaque, Southern Pig-tailed Macaque, Squirrel Monkey, Vervet Monkey	
Animal, Instrument, and Surgeon Preparation	

1. **Animal Preparation including hair / fur removal, disinfection of operative site and draping operative site with sterile material**
   The surgical site is shaved. A sterile prep using ChlorPrep (a chlorhexidine/ alcohol instant solution) is be applied to the entire surgical site and allowed to dry.

2. **Sterilization of Surgical Instruments**
   Instruments are sterilized using a steam autoclave or ethylene oxide gas.

3. **Surgeon Preparation and PPE**
   All surgical staff dons hair bonnets, surgical masks, protective eyewear and gloves prior to entering the operating room. The surgeons perform full presurgical sterile hand scrubs with a chlorhexidine or betadine solution (if indicated based on procedure being performed). Alternatively, presurgical Avaguard is applied to both hands for sterile prep. The surgeons don sterile gowns (if indicated), and sterile surgical gloves prior to draping the patient and beginning the surgical procedure.

4. **Animal Support including eye lubrication, heat support, and fluid support**
   Eyes will be lubricated, and a continuous source of warmth will be provided. Fluid support for many surgical procedures is provided via intravenous catheter. Isotonic crystalloid solutions may be administered typically at a rate of 5-10 ml/kg/hr as directed by veterinary staff.

1. **Post-surgical care including recovery from anesthesia, removal of sutures or wound clips, and monitoring incisions for post-surgical infections.**
   Post-surgical care will follow the anesthesia recovery standards outlined in GL-057 and GL-075 and the surgical procedure recovery standards described in SOP SR-020.

2. **Criteria for monitoring pain and distress including the frequency and duration of monitoring for short and long term post-operative assessment plans.**
   Post-operative monitoring and assessment of pain and distress will be accomplished by surgical veterinary staff (Surgery SOP SR-020 and Guideline GL-016) typically for a minimum of 3-7 days.

3. **Clinical signs used to determine whether animals need veterinary care or should be euthanized.**
   Observation for species specific behaviors, food and water consumption, urine and feces production, general demeanor, incision healing and presence of pain will be performed daily. Any abnormalities will be reported to the surgical veterinarians.

4. **Post-operative prophylactic antibiotics**
   Post-operative antibiotics may be prescribed at the discretion of the surgical veterinarians in consultation with the research staff.

5. **Post-operative analgesia to prevent or relieve pain and distress**
   Post-operative analgesia will be provided for 2 days following the surgical procedure using buprenorphine 0.01-0.1 mg/kg, administered intramuscularly, twice a day (typically between 7:30-8:30 am and 7:30-8:30 pm). Non-steroidal anti-inflammatory drugs may be added to the analgesia regime at the discretion of the surgical veterinarians after consultation with the investigative staff. If sustained release buprenorphine is administered pre-op, no additional opioids will be given post-op unless breakthrough pain is noted.

1. **Description of Euthanasia Method**

   Nonhuman primates will be sedated with 20 mg/kg of ketamine (or other anesthetic d r u g at appropriate dosage at veterinary discretion) and transported to a necropsy suite. Animal is then anesthetized with sodium pentobarbital administered intravenously at 25 mg/kg. Depth of anesthesia is assessed by loss of palpebral, corneal, withdrawal and gag reflexes, followed by exsanguination via the distal aorta (ref. SOP PT-001 *Nonhuman Primate Necropsy*; GL-076 *Guidelines for NHP Euthanasia*).

2. **The euthanasia method is acceptable under the AVMA guidelines for the Euthanasia of Animals.**

BACKGROUND:

During post-procedural and post-operative periods, animals may experience pain and are more vulnerable to environmental stressors. Frequent and careful observations by qualified personnel are critical to identify pain or distress. The objective is to minimize pain and distress with consideration for the scientific integrity of the research.

SCOPE:

This policy applies to any animal used in teaching or research at OHSU that undergoes survival surgery or an experimental procedure. This policy applies to both acute and long term post- surgical and post-procedural care.

POLICY:

All post-operative/procedural care must be described in the animal use protocol and approved by the IACUC. The description must include analgesia and nursing care as appropriate. Animals must be observed for signs of pain or distress and monitored to ensure normal physiological functions and behavior. Exceptions to this policy may be granted by the IACUC based on scientific justification for withholding post-operative/procedural pain medication.

PROCEDURES:

I. Post-operative and post-procedural care will be provided to the animal until it is fully recovered. The animal is considered fully recovered when it is eating and drinking normally and in the case of post- surgical recovery, incisional wound healing is complete and, when applicable, sutures are removed.

II. Any suspected complication, pain, or discomfort will be brought to the attention of the veterinary staff for immediate consultation.

III. It is the investigator's responsibility to assure that appropriate post-surgical/procedural observations are made and documented, and that the animal receives appropriate care during the post-surgical and post-procedural recovery unless a veterinarian takes responsibility for the case.

IV. Appropriate record keeping which documents animal recovery is required for USDA covered species and is suggested for non-USDA covered species.

V. Post-operative procedures for nonhuman primates on West Campus are delineated in the Postoperative Care standard operating procedure SR-034 Postoperative Care, GL-075 Guideline for Anesthesia in NHPs.

VI. For Central and Waterfront Campuses and for rodents and rabbits on West Campus, the procedures outlined below should be followed.

A. Veterinary Oversight

i. Post-operative and post-procedural care are considered to be an aspect of adequate veterinary care.

ii. DCM veterinary staff will be available to assist investigators in planning post-operative or post procedural care.

iii. The veterinary staff should observe the post-operative or post-procedural area as often as necessary to ensure adequate veterinary care.

B. Principal Investigator and Scientific Staff Responsibilities

i. Surgical or Anesthetic Recovery (acute)

a. The need for monitoring will vary according to the type of surgery performed, anesthetic that was used, and species of animal.

At a minimum, recovery records should indicate the time at which the animal was examined, the person who examined the animal, and any abnormal findings. Surgical and/or anesthetic records should be available for veterinary or IACUC review.

ii. Animals are categorized in a certain stage of recovery as follows:

a. Stage 4: Animals are unconscious or semi-conscious, unable to sit up or maintain sternal recumbency. Most reflexes will be absent or severely diminished. Assessment of physiologic parameters should continue as was done during the surgical/anesthetic procedure.

b. Stage 3: Animals are semi-conscious, but aroused when handled. Typically they will not be able to control body position. Return of reflexes continues and endotracheal tubes (if used) are usually removed in this stage when the gag/swallow reflex is present.

c. Stage 2: Animals can, at minimum, maintain themselves in a sternal position. They may be able to stand or ambulate but show signs of sedation or ataxia.

d. Stage 1: Animals function normally without any discernible effects from anesthetic event (unless directly altered by experimental procedure).

iii. Animals should never be left unattended until after extubation (if applicable) and sufficient recovery (stages 2-3).

iv. The following parameters (as applicable by species) should be observed until the animal recovers to Stage 2 (when the animal is extubated and able to reach food and water):

a. Stage 4 monitoring (every 15-30 min)

1. Body temperature
2. Heart rate and pulse character
3. Respiratory rate and pattern
4. Capillary refill time
5. Jaw tone - resistance or no resistance to opening
6. Response to toe pinch - withdrawal or non-withdrawal
7. Palpebral response to touching the medial canthus
8. Color of mucous membranes at gums or conjunctiva
9. Examine closely for other abnormalities

b. Stage 3 monitoring (every1 hour)

1. Body temperature
2. Capillary refill time
3. Color of mucous membranes at gums or conjunctiva
4. Attitude and activity
5. Condition of the operative site
6. Examine closely for other abnormalities

v. Extubation should be performed when swallowing reflex has returned. Time of extubation should be recorded.

vi. Animals should be turned from side to side periodically to help prevent pulmonary hypostatic congestion and other complications.

vii. Ambient temperature should be adjusted by increasing room temperature or cautiously using circulating warm water blankets or heat lamps (close monitoring is required when using heat lamps to prevent heat stress). The animal should be kept dry

and free of excreta and drapes or towels should be placed between the animal and cold surfaces. In addition, post-op fluids may be warmed prior to administration, especially in small animals. Electric heating pads must not be used in direct contact with an animal.

viii. Careful attention should be placed on assessing pain and distress in the animals during recovery from anesthesia, especially for major surgeries or any procedure likely to cause pain. Administration of analgesics must be provided as described in the approved protocol or at the discretion of the veterinary staff.

ix. Hydration should be assessed and fluid replacement administered at a volume of 10-40 ml/kg body weight for animals that are not eating and drinking post-operatively/procedurally. Fluids may be given parenterally, either subcutaneously, intraperitoneally or intravenously (IV rate of 10-40 ml/kg/h in larger animals). Lactated ringers solution or equivalent crystalloid should be utilized.

x. Animals can be returned to their housing area when they are sternal and able to ambulate in a manner that allows them to safely obtain food and water.

xi. Surgical or Anesthetic Recovery (long-term)

a. General well-being of the animal should be monitored daily and the post-surgical/anesthetic record continued at a frequency appropriate for the procedure (until removal of sutures if surgery was performed) and should include the following:

a. Visual confirmation of eating and drinking

b. General appearance of animals (e.g. sunken appearance, rough hair coat)

c. Attitude and activity (e.g. alert or lethargic)

d. Presence of stool or urine

e. Condition of operative site if applicable (e.g. wound healing, dehiscence, etc.)

f. Any other notable abnormalities (related or unrelated to the procedure)

b. Continue assessment of pain and distress with a frequency appropriate for the procedure and possible complications associated with it. Consultation with the veterinarians may help determine the best post-operative pain monitoring schedule when writing a protocol.

c. Any concerns with the well-being of the animal during the post-procedural/surgical monitoring period should be brought to the attention of the veterinary team to determine if additional care is required.

REFERENCES:

AWA Regulations 2.31,d,

Guide for the Care and Use of Laboratory Animals (Guide) 8th ed, (2011)

Office of Laboratory Animal Welfare IACUC Guidebook (OLAW) 2nd ed, (2002)

Policy Title: Non-human Primate Environmental Enhancement to Promote Psychological Well-being	Program: Animal Care and Use
Policy Owner: Integrity Department	Date Effective: 12/19/2018

BACKGROUND:

Environmental enrichment of non-human primates (NHPs) is required by the AWA and the Guide for the Care and Use of Laboratory Animals, and includes social housing and enhancement to the animal's environment.

SCOPE:

This policy applies to all non-human primates (NHP) at OHSU facilities.

POLICY:

NHPs must be socially housed with compatible conspecifics whenever possible. Pair housing is the minimum goal. NHPs must also be provided with various forms of environmental enrichment.

PROCEDURES:

I. NHPs can be excluded from social housing for medical or behavioral reasons, or for scientifically justified reasons approved by the IACUC.

II. Single housing of NHPs assigned to research protocols must be reviewed by the Attending Veterinarian on Central and Waterfront Campus or the NHP Behaviorist on West Campus prior to IACUC approval.

III. Special attention must be afforded to single housed animals to maintain psychological well-being. Single housed animals should have tactile, visual and/or auditory contact with conspecifics.

IV. The environment of each caged NHP should be enriched with cage furniture (e.g., perches) and with different manipulanda. . Enrichment enhancements may include any or all of the following: foraging devices or toys in the primary housing enclosure; cage furniture such as perches for caged animals and swings and climbing devices for group housed animals; television or radio; positive reinforcement training; and/or cognitive enrichment (e.g., tablets). Enrichment items may be rotated to prevent habituation.

V. Restriction from specific enrichment enhancements for valid scientific reasons requires IACUC approval. Enrichment may also be restricted for clinical and/or behavioral reasons.

VI. All new non-human primate protocols will be reviewed by the IACUC for compliance with this policy. Exemptions from social housing will be reviewed by the Attending Veterinarian on a monthly basis and by the IACUC quarterly.

AUTHORITY:

AWA Regulations

Guide for the Care and Use of Laboratory Animals

REFERENCES:

ONPRC Non-Human Primate Behavioral Management Plan

Environmental Enrichment for Marmosets, Central and Waterfront Campus
